# On brain stimulation in epilepsy

**DOI:** 10.1093/brain/awae385

**Published:** 2025-01-02

**Authors:** Andrew J Trevelyan, Victoria S Marks, Robert T Graham, Timothy Denison, Andrew Jackson, Elliot H Smith

**Affiliations:** Newcastle University Biosciences Institute, Newcastle upon Tyne, NE2 4HH, UK; Institute of Biomedical Engineering, Oxford University, Oxford, OX3 7DQ, UK; Institute of Neurology, University College London, Queens Square, London, WC1N 3BG, UK; Institute of Biomedical Engineering, Oxford University, Oxford, OX3 7DQ, UK; Newcastle University Biosciences Institute, Newcastle upon Tyne, NE2 4HH, UK; Department of Neurosurgery, University of Utah, Salt Lake City, UT 84132, USA

**Keywords:** epilepsy, seizure, optogenetics, brain-machine interface, neuromodulation, feedback control

## Abstract

Brain stimulation has, for many decades, been considered as a potential solution for the unmet needs of the many people living with drug-resistant epilepsy. Clinically, there are several different approaches in use, including vagus nerve stimulation, deep brain stimulation of the thalamus, and responsive neurostimulation. Across populations of patients, all deliver reductions in seizure load and sudden unexpected death in epilepsy risk, yet do so variably, and the improvements seem incremental rather than transformative. In contrast, within the field of experimental neuroscience, the transformational impact of optogenetic stimulation is evident; by providing a means to control subsets of neurons in isolation, it has revolutionized our ability to dissect out the functional relations within neuronal microcircuits. It is worth asking, therefore, how preclinical optogenetics research could advance clinical practice in epilepsy?

Here, we review the state of the clinical field, and the recent progress in preclinical animal research. We report various breakthrough results, including the development of new models of seizure initiation, its use for seizure prediction, and for fast, closed-loop control of pathological brain rhythms, and what these experiments tell us about epileptic pathophysiology. Finally, we consider how these preclinical research advances may be translated into clinical practice.

## Teaser

Many people living with epilepsy continue to have seizures despite medical treatment, and for these patients, brain stimulation devices may be considered. Trevelyan *et al.* review the current use of brain stimulation in epilepsy, and consider how recent advances in preclinical research could be translated into clinical practice.

## Introduction

Animal models of neurological disease can seem far removed from clinical practice, particularly when the experiments involve novel research techniques. The continued utility of animal models, though, derives from the fact that many experimental techniques, particularly at the cellular or small network level or involving transgenic manipulations, are unavailable for human use, when they are readily implemented in animals. The development of new research techniques and technology often provides huge impetus towards understanding complex facets of biology, and one of the tenets of modern medicine is that understanding leads to better healthcare.

Seizures are the most intense level of brain activation, and consequently, they represent a particular challenge for novel neurotechnology. Currently, just three brain stimulation devices are clinically approved for treating epilepsy: vagus nerve stimulation (VNS), responsive neurostimulation (RNS) and deep brain stimulation of the anterior nucleus of the thalamus (ANT). All use electrical stimulation, but optogenetics, which has become a mainstay in basic neuroscience research, offers something fundamentally different.^1,2^ Electrical stimulation activates neurons according to their relative excitability and proximity to the electrode; in contrast, using optogenetics, one can target cells selectively, either exciting^3^ or inhibiting^4^ them, regardless of their intrinsic excitability or location. This is achieved by the highly restricted localization of opsins, light-sensitive membrane proteins that act as either ion channels or pumps. Since only cells expressing the opsin are sensitized, only they are directly affected when the tissue is bathed in light.

The clinical implementation of optogenetics would still involve an implanted device, but also the considerable additional hurdle of requiring gene-editing within the brain.^5-7^ This is the key difference from the research field, which benefits from excellent transgenic animal lines and effective viral vectors, leading to advances in our understanding of epileptic pathophysiology, while indicating new possibilities for controlling epileptic activity that may still be achieved using electrical stimulation.

## Using stimulation to control epilepsy: neuromodulation or ‘neuroversion’

There is a huge unmet clinical need in epilepsy for new kinds of therapy: ∼30% of epilepsy cases are medically refractory,^8^ and while surgical treatment options are available, these are under-utilized and the patient path towards these are slow,^9^ reflecting both perceived and real challenges.^10,11^ Resective surgery can be curative, but is contraindicated in many cases,^9^ for instance, if an epileptic focus cannot be identified, or is too large or too distributed, or involves eloquent cortex (areas that subsume speech or important motor or cognitive functions). Brain stimulation, however, may be used in such cases, because unlike resective surgery, relatively minimal permanent damage is incurred (although any penetrative implant will cause some damage), and the rate of adverse effects is low (∼4%–10% of cases^12,13^; higher numbers report chronic neuropsychiatric issues, including depression, but these likely reflect the persistence of pre-implantation rates^12^). Furthermore, once devices are *in situ*, stimulation may be delivered in different ways, to achieve a range of effects. Thus, we see an expansion of surgical treatment options, and also their potential application in cases that previously may not have been considered for surgery.

Given the proven worth of cardioversion, the idea that seizures may be similarly terminated by electric shocks is attractive. Ideally, one could record brain activity to detect seizures at their onset, or even better just beforehand, and then intervene to stop them. This is termed RNS or ‘closed-loop’ control. The alternative is to deliver stimulation continually, termed open-loop, which is how both VNS and ANT stimulation is done. Notably, all these stimulation devices deliver significant reductions in seizure load relative to pre-implantation (or to epochs of no stimulation) and typically show lower rates of sudden unexpected death in epilepsy (SUDEP) compared with other cohorts of drug-resistant epilepsy.^12,13^ The SANTE trial, a multi-centre, double-blind trial of bilateral ANT stimulation (110 cases, followed over 10 years now) showed a 75% reduction in seizure load,^12^ similar to outcomes from VNS or resective surgery.^13^ The precise location of stimulation electrodes in RNS appears not to be a good predictor of outcome^14^; good outcomes were still possible even if the seizure foci were poorly defined after intracranial monitoring,^15^ although cortical stimulation can outperform subcortical stimulation.^13^ RNS devices that were reprogrammed to deliver low frequency (<10 Hz) open-loop stimulation achieved at least as good, if not better, seizure reduction (76%, compared to 13% during the RNS epoch, although not significantly different, due to high variance and the low sample size^16^). Interestingly, RNS outcomes were better when more of the stimulation occurred during apparently ‘low risk’ periods,^17^ suggesting that their main benefit was not from rapid closed-loop control. In this study, poor responders often had periods of stimulation that were phase-locked to high amplitude, pathological discharges, without ostensible benefit.^17^ Finally, in this brief synopsis of the clinical data, seizure reduction tended to happen rather slowly, over months to years.^12,18,19^ In summary, ‘neuroversion’ of seizures has yet to be convincingly demonstrated using any clinically available device, suggesting that clinical benefits arise primarily from neuromodulation.^20^

One factor that may confound attempts to terminate seizures using stimulation is that, during seizure propagation, discharges arise from the edge of the expanding seizure.^21,22^ Consequently, once a seizure starts to spread, the target area for controlling it becomes ever larger and more distributed, both factors that make control more difficult to achieve from focal stimulation, especially in larger brains. Focal stimulation control may therefore only be possible very early in a seizure, or even better, if seizure prediction can be improved, ahead of it.

Despite the underwhelming clinical data, animal studies suggest a more optimistic outlook: several research groups have independently provided direct proof-of-principle demonstrations of closed-loop seizure termination in different animal models.^23-27^ Interestingly, seizure termination is possible using stimulation at sites (the subiculum,^27^ thalamus,^24^ the medial septum connecting the hippocampi^28^ and even the cerebellum^25^) well removed from the pathological foci. These sites may represent bottlenecks within the epileptic network, or instead, be well connected sites capable of suppressing activity elsewhere in the brain.^29^ Such ‘hubs’ in the epileptic network represent ideal sites both for monitoring pathological activity or for targeted interventions, even if the true seizure source cannot be identified. The primary message from these preclinical studies is an optimistic one, that there remains plenty of room for improvement upon best current clinical practice.

It is appropriate, therefore, to ask what is different in these animal studies? Several factors suggest themselves. First, experimental models tend to have better defined pathology, with less variability, than clinical cases. Second, most of these studies were done in mice, where stimulation may influence a relative greater extent of the network (although others use non-human primates^23^). A common theme across these studies is that they all used optogenetic stimulation. Together with the benefits mentioned earlier, optogenetic stimulation does not, itself, generate an electrical signal (as long as the electrode is not illuminated directly), making it particularly suitable for instantaneous feedback (‘closed-loop’) control.^23^ In contrast, electrical stimulation inevitably generates a large electrical artefact, which needs to be ‘blanked out’ in feedback systems, often with several milliseconds on either side of the artefact. Clearly, optogenetics has facilitated closed-loop control in animal models of epilepsy, but an unresolved question is whether, in an optimized animal model, electrical stimulation in some form could achieve as good results as optogenetic stimulation; this study is yet to be done but is critical if one were to make the case for optogenetics, given the additional challenges to clinical implementation.

Animal studies have also demonstrated anti-epileptic effects through continual, open-loop, optogenetic stimulation, at a surprisingly wide range of frequencies, from 100 to 0.033 Hz,^30-34^ but notably, the effect varies at different frequencies of stimulation.^30^ Interestingly, interictal epileptiform discharges also reduce the likelihood of tonic-clonic seizure-like discharges,^35-37^ suggesting that internally generated, spontaneous discharges are, in this respect, equivalent to brain stimulation. Given the inherent risks of stimulating a hyperexcitable brain, it is reassuring to realize that brain stimulation can be anti-epileptic. The reduction in network excitability induced by repeated stimulation is a form of homeostatic neuromodulation, which is, in fact, predicted on theoretical grounds.^38^ On the other hand, stimulation when the state is close to its tipping point (also known as its ‘critical threshold’) can also push the system into the pathological state.^39^ A key issue, therefore, is to determine what proportion of time the network lies close to that critical threshold, and to adapt the stimulation strategy accordingly.

Homeostatic neuromodulation may occur at multiple different time scales, and by extension, the different frequency effects may be mediated by different systems. High frequency stimulation, as used for deep brain stimulation, most likely works by rendering key neurons perpetually refractory, effectively inhibiting them^40^; lower frequency stimulation may work instead by triggering different molecular regulators of excitability, such as metabotropic glutamate receptors,^41,42^ endocannabinoids^43^ or the MAPK pathway,^44,45^ among others. While cannabinoids are an active area of epilepsy therapeutics research, the others are not. The optogenetic neuromodulation of animal seizure models offers an attractive new assay for pharmacological investigations that can advance this area of research, indicating novel ways to treat the condition.

## Using stimulation for seizure prediction

**Figure 1:**
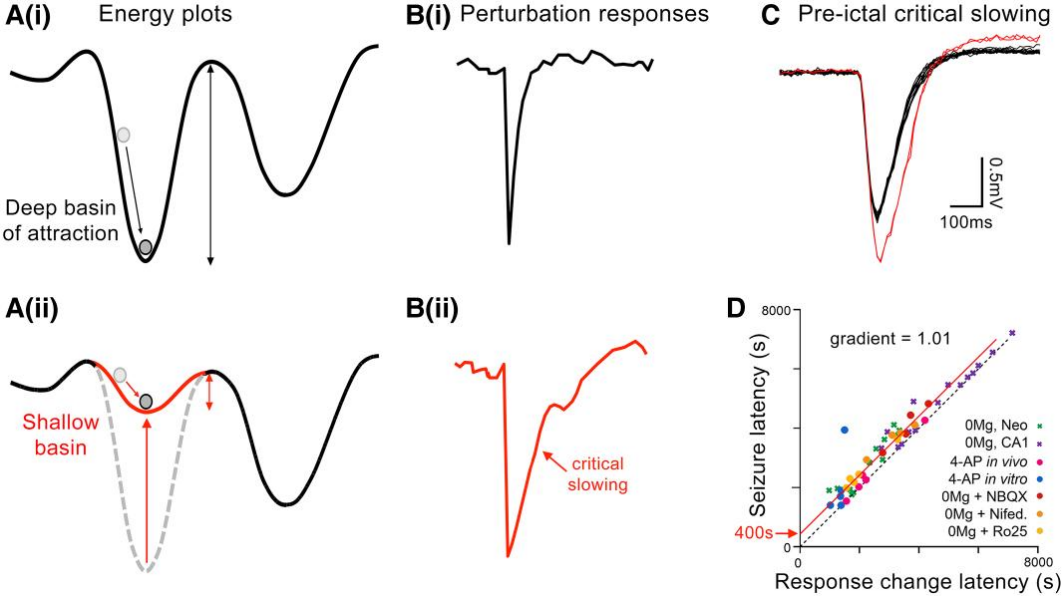
**‘Critical slowing’ predicts the transition into seizure activity**. (**A**) Schematic representations of the energy of a dynamic system (adapted from Scheffer *et al*.^46^). Perturbations may transiently knock the system from its stable position at the trough of a ‘basin of attraction’, but the system rapidly returns to that state (**B**). For shallower basins [**A**(**ii**)], the force towards the valley is weaker and so the recovery from a perturbation is slower [**B**(**ii**)]. The transition to a different state requires a perturbation of sufficient energy to overcome the energy barrier between the states (double-headed arrows in **A**); this is obviously easier to achieve for shallower basins, which are thus said to lack resilience. (**C**) The black traces show 12 consecutive responses to an optogenetic stimulation of pyramidal cells, followed by two further responses immediately after, showing the same critical slowing in the response, immediately prior to the onset of seizure-like activity, in a mouse brain slice. (**D**) The timing of the critical slowing transformation showed a highly significant correlation with the onset of seizures, in a range of acute pharmacological models of ictogenesis.^19^ Notably, this plot had a unitary gradient (indicative of a precise temporal correspondence of the two changes) and an intercept of ∼400 s, which is the average advance warning ahead of the seizure onset provided by this biomarker.

Another use of brain stimulation that remains underexplored clinically, is for assaying brain states. This strategy is motivated by the key insight that various different complex systems, including climate, ecological, biological and financial, share common dynamical features, which may be revealed by perturbing them. A useful metaphor is a landscape, where the energy of a system is conceptualized as a set of valleys, separated by peaks. These valleys, or basins of attraction (hence, the term ‘attractors’), are where the system might settle; the steeper the valley, the more entrenched is the state (Fig. 1A). If one challenges the system (a ‘perturbation’), it quickly re-settles into the bottom of the valley again [Fig. 1A(i)]. If, however, the valley becomes shallower, then the recovery to that stable minimum is slowed [Fig. 1A(ii)]. A progressive move towards shallower valleys requires less energy to transition into a different valley (a state change, or tipping point); the slowing of recovery from a perturbation is a means of assessing the shallowness of the valley. Thus derives the term ‘critical slowing’, describing the system as it approaches a ‘critical threshold’ or ‘tipping point’ (also referred to as a ‘catastrophic bifurcation’). The proximity to the tipping point is also evident from other features of the system,^46^ for instance, in a higher lag-1 autocorrelation^39,47^ or increased variance.^39,48^ Interestingly, features of these various systems have been reported in analyses of human electrographic recordings associated with imminent seizure onset,^39,49,50^ although little yet from the analysis of perturbations. The one notable attempt to do this took its lead from the pattern of flickering light used to assess photosensitivity^51^ and examined the response to 10–20 Hz stimulation delivered to the hippocampus in six patients.^52^ They reported that a measure of the spectral properties of the local field potential response was relatively higher at sites close to the epileptic focus, and also increased in the few hours prior to a seizure.^52^ A closely related approach is to use stimulation to map cortical territories, spatially,^53^ rather than temporally. When mapping the spatial structure of the pathology, it can be helpful even to trigger seizures, in the safety of the epilepsy monitoring unit. One such study found that 50 Hz stimulation triggered ‘patient-typical’ seizures in 70% of cases; more unusual were cases when these seizures were triggered by 1 Hz stimulation, but these turn out to be the best indicators of the seizure onset zone, presumably reflecting the fact that they are peculiarly excitable sites.^54^

If, on the other hand, one wants to monitor dynamic shifts in seizure susceptibility, rather than the spatial structure, using a 20 Hz train of stimuli^52^ runs too much risk of actually triggering a seizure. It is extremely encouraging therefore that several recent animal studies indicate that single, transient optogenetic stimuli can be used to monitor local network excitability,^30,55,56^ yet do not trigger seizures themselves.^30^ In these studies, selective stimulation of excitatory neurons elicited a response that showed an all-or-nothing change, as network excitability increases, indicative of some threshold having been surpassed (Fig. 1B).^30,55,56^ Optogenetic stimulation of different interneuron populations, on the other hand, only showed altered responses after the seizure had started.^30^ Of key importance, the sudden change in the response to pyramidal stimulation showed a precise temporal correspondence to the time when seizure activity started (Fig. 1C),^30^ indicative of a strong causal link. Notably, the change in response always occurred in advance of seizure initiation, by several hundred seconds, sufficient time to enact some kind of intervention (Fig. 1D). One final point that deserves comment is that the change in the response was indicative of a dendritic action potential (a ‘plateau potential’)^30^ and this has a striking parallel with the Valentin *et al*.^57^ report that single pulse electrical stimulation, on occasions, identified sites with sustained (100–1000 ms) evoked responses, and that surgically removing those sites was associated with far better post-surgical outcomes. For more information about clinical stimulation studies, we direct readers to two recent reviews from the recent ICTALS conference.^58,59^

## Using stimulation to advance our understanding of (patho)physiology

**Figure 2:**
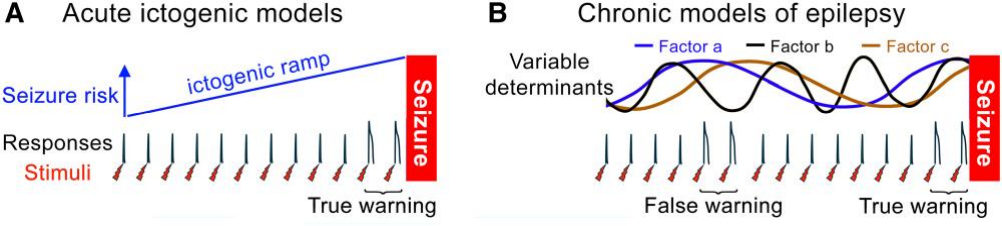
**Chronic models of epilepsy are likely to yield weaker correlations between critical slowing and seizure onset**. (**A**) Schematic representation of an acute model of ictogenesis, in which a pharmacological manipulation creates an ‘ictogenic ramp’, driving the system monotonically towards seizures. Note the change in response prior to the seizure onset (arbitrary time scale). (**B**) Similar schematic of a chronic model of epilepsy, in which multiple factors interact, to create a variable seizure risk. These are presumed to summate, although the summation is unlikely to be linear, to create a multifactorial risk, which may be mapped less precisely than for the simple acute models. For instance, there may be times when the critical slowing transition occurs (change in responses marked ‘False warning’), but if the system then retreats from seizure threshold without a seizure actually occurring, the perturbations would give a false-positive warning. Consequently, chronic models may yield less precise correlations between critical slowing and seizure predictability than the acute models (Fig. 1D).

The aforementioned optogenetic studies were all performed using acute pharmacological models of seizure induction^30,55,56^ and have yet to be replicated in chronic models of epilepsy, but clearly that is the next step towards clinical implementation. It is helpful therefore to consider briefly what such a study may look like. Acute ictogenic models drive the network inexorably towards the seizure state, creating what might be termed an ictogenic ramp (Fig. 2A). As such, these models lend themselves to identifying, and characterizing, biomarkers for a tipping point in the system. In contrast, chronic models, which may be closer to the real clinical scenario, show a waxing and waning pattern of seizure risk, influenced by multiple factors that may not necessarily be correlated (Fig. 2B). Consequently, even if one optimizes the brain stimulation parameters and the location of the stimulation and recording sites, the results could still be less precise than for a simple ictogenic ramp; in the chronic epileptic state, there will be false positives, when the stimulation response indicates a pre-ictal state, yet the system then settles down without a seizure occurring (Fig. 2B). These false positives will inevitably compromise the degree of correlation between the stimulation response change and the onset of seizures. This again, is an argument for starting simple and then building up the complexity of experimentation, to avoid missing promising leads.

While the primary clinical interest is to develop anti-epileptic stimulation protocols, optogenetic stimulation may also be used to trigger or escalate pathological discharges. These novel experimental models shed new light upon how both seizure^30,60-64^ (reviewed elsewhere^65^), and cortical spreading depression events may start^66^ (the latter may be relevant also for seizure termination). A key insight has been to identify the various negative feedback mechanisms that stabilize physiological brain states, and other positive feedback mechanisms that operate synergistically to push the network towards a self-organizing epileptic state.^30,60^ The synergy between the positive feedback mechanisms may help explain the precipitate nature of seizure onset.

These forces are often scaled with respect to distance from the initial stable state (physiological baseline). The general principles of attractor dynamics tell us that a tipping point occurs when the centrifugal forces (pushing away from the starting point) exceed the centripetal ones (pushing back—negative feedback). This may happen if the centripetal forces weaken with distance, or if the centrifugal force increases (positive feedback). Interestingly, studies of other dynamical systems indicate that positive feedback mechanisms may be the defining feature of many systems that show critical state changes.^46,67,68^

The large change in the postsynaptic glutamatergic response that occurs prior to the onset of epileptic discharges^30^ closely resembles the appearance of critical slowing. Its all-or-nothing nature provided a strong clue that we were, in fact, recording dendritic spikes,^69^ mediated by NMDA receptors or voltage-gated Ca^2+^ channels (VGCCs); this was confirmed using Ca^2+^ imaging.^30^ Both NMDA receptors and VGCCs, like Na^+^ channels, are depolarizing conductances that are activated by depolarization, and so constitute positive feedback mechanisms, an effect that is further enhanced by triggering bursts of somatic action potentials, which delivers more glutamatergic drive back into the local network. Interestingly, the generally accepted ionic conductance model of the paroxysmal depolarizing shift is based upon these same slow kinetic VGCCs,^70-72^ while multiple other studies have also found associations between changes in dendritic excitability, neuronal burst firing and epileptic susceptibility.^73-78^ Other contributory positive feedback effects include ionic redistribution patterns that are caused by neuronal activity and facilitate further neuronal activation: rises in intracellular [Cl^−^], which reduces inhibitory synaptic efficacy, and extracellular [K^+^], which brings neurons closer to their action potential thresholds. Note, however, that other ionic changes, such as depletion of extracellular Na^+^ and Ca^2+^, reduce network excitability, and so should be considered to be negative feedback. Together with the homeostatic effects mentioned earlier, collectively, we are building a picture of multiple different negative and positive feedback mechanisms involved in this critical transition, although this is unlikely to be a comprehensive list; other rapidly acting feedback effects may be mediated by I_h_,^76,77^ GABA_B_,^35,36^ I_NAP_^79^ and more besides.

Brain activity shows regular transitions as part of a natural (and healthy) circadian cycle, and interestingly, dendritic plateau potentials have been implicated in other transitions of cortical network function, for instance underlying perceptual thresholds.^80^ This is a further example of a fundamental cortical function that appears to carry the inherent risk of escalating network activation.^81^ On a more practical level, it raises another issue, which is that any seizure prediction algorithm based upon detecting critical slowing will need to distinguish between imminent physiological or pathological state changes.

## Future translation

Brain stimulation, in its various forms, has been used to treat the most difficult epilepsy cases for many decades now, and while these cases generally show improvement, the outcomes are rarely transformative. Recent animal work, however, indicates its potential, begging the question how we build upon these preclinical results. Using brain stimulation for seizure prediction has not been properly tried in humans, but this is an obvious early goal. Given that open-loop stimulation appears relatively safe and may even be anti-ictogenic in its own right, we should not be shy to expedite these clinical studies. An important question, however, is whether clinical electrodes, as they are currently designed, are up to the task of delivering the appropriate stimulation, or record with sufficient precision to be able to detect the changes that have been shown using optogenetics in animals. The critical biomarker in mice reflects the threshold for triggering dendritic spikes in populations of pyramidal cells. These plateau action potentials yield clear and measurable change with a high signal-to-noise ratio in mice. As such, they are attractive candidates for a clinical-grade sensor, but to optimize the chance of a successful clinical trial, the design of the sense-stimulation electrodes should aim to maximize the signal-to-noise. The translational path is likely to involve new electrode and algorithm designs, implemented within a modular framework,^82,83^ that should also be tested in animals in which the relevant neuronal structures (e.g. cortical thickness) are comparable to our own.

The characterization of dendritic excitability may also help establish the parameters of stimulation in patients in the clinic, by providing an easily measured frame of reference. To explain this point, it is helpful to consider another neurological condition for which brain stimulation has been used very successfully: in Parkinson’s disease, the target site for stimulation is reasonably well defined and the parameter space for stimulation (e.g. frequency and amplitude of stimulation) can be explored very quickly, because the effects on a patient’s tremor are apparent immediately. By contrast, epilepsy is a more complex and heterogeneous pathology, and the clinical benefits are only measured over many months. A rapid means of calibrating the stimulation parameters, for instance, using the threshold for dendritic spikes, would help greatly. It may also help determine where to stimulate, which patients are likely to respond to treatment^84^ and finally, provide the means to compare patient outcomes.
